# The cortical microstructural basis of lateralized cognition: a review

**DOI:** 10.3389/fpsyg.2014.00820

**Published:** 2014-07-30

**Authors:** Steven A. Chance

**Affiliations:** Neuropathology, Nuffield Department of Clinical Neurosciences, Neuroanatomy and Cognition Group, University of OxfordOxford, UK

**Keywords:** minicolumn, cytoarchitecture, lateralization, asymmetry, face-processing, language, schizophrenia, autism

## Abstract

The presence of asymmetry in the human cerebral hemispheres is detectable at both the macroscopic and microscopic scales. The horizontal expansion of cortical surface during development (within individual brains), and across evolutionary time (between species), is largely due to the proliferation and spacing of the microscopic vertical columns of cells that form the cortex. In the asymmetric planum temporale (PT), minicolumn width asymmetry is associated with surface area asymmetry. Although the human minicolumn asymmetry is not large, it is estimated to account for a surface area asymmetry of approximately 9% of the region’s size. Critically, this asymmetry of minicolumns is absent in the equivalent areas of the brains of other apes. The left-hemisphere dominance for processing speech is thought to depend, partly, on a bias for higher resolution processing across widely spaced minicolumns with less overlapping dendritic fields, whereas dense minicolumn spacing in the right hemisphere is associated with more overlapping, lower resolution, holistic processing. This concept refines the simple notion that a larger brain area is associated with dominance for a function and offers an alternative explanation associated with “processing type.” This account is mechanistic in the sense that it offers a mechanism whereby asymmetrical components of structure are related to specific functional biases yielding testable predictions, rather than the generalization that “bigger is better” for any given function. Face processing provides a test case – it is the opposite of language, being dominant in the right hemisphere. Consistent with the bias for holistic, configural processing of faces, the minicolumns in the right-hemisphere fusiform gyrus are thinner than in the left hemisphere, which is associated with featural processing. Again, this asymmetry is not found in chimpanzees. The difference between hemispheres may also be seen in terms of processing speed, facilitated by asymmetric myelination of white matter tracts (Anderson et al., 1999 found that axons of the left posterior superior temporal lobe were more thickly myelinated). By cross-referencing the differences between the active fields of the two hemispheres, via tracts such as the corpus callosum, the relationship of local features to global features may be encoded. The emergent hierarchy of features within features is a recursive structure that may functionally contribute to generativity – the ability to perceive and express layers of structure and their relations to each other. The inference is that recursive generativity, an essential component of language, reflects an interaction between processing biases that may be traceable in the microstructure of the cerebral cortex. Minicolumn organization in the PT and the prefrontal cortex has been found to correlate with cognitive scores in humans. Altered minicolumn organization is also observed in neuropsychiatric disorders including autism and schizophrenia. Indeed, altered interhemispheric connections correlated with minicolumn asymmetry in schizophrenia may relate to language-processing anomalies that occur in the disorder. Schizophrenia is associated with over-interpretation of word meaning at the semantic level and over-interpretation of relevance at the level of pragmatic competence, whereas autism is associated with overly literal interpretation of word meaning and under-interpretation of social relevance at the pragmatic level. Both appear to emerge from a disruption of the ability to interpret layers of meaning and their relations to each other. This may be a consequence of disequilibrium in the processing of local and global features related to disorganization of minicolumnar units of processing.

The significance of human brain asymmetry depends broadly on two lines of evidence: the presence of anatomical asymmetries at the large and small scale and the presence of functional lateralization of cognitive functions, most notably language. A major challenge is that the nature of the link between the two is not clear. For example, the simplest models tend to be based on the principle that a larger brain region on one side of the brain denotes dominance for a lateralized function (Galaburda, 1995). However, there are frequently exceptions to this rule. Asymmetries vary by degree between individuals. Furthermore, the correspondences between structures within the same individual and between structural asymmetry and functional lateralization are often inconsistent.

## AUDITORY CORTEX, LANGUAGE, AND ASYMMETRY

In humans, the superior temporal gyrus (STG) contains perhaps the most prominently asymmetrical brain area: the auditory association cortex of the planum temporale (PT), lying posterior and lateral to Heschl’s gyrus, contributing to the hemispheric asymmetry of the posterior Sylvian fissure. This region plays a key role in phonological processing and forms part of the receptive language region often identified as Wernicke’s area. Geschwind and Levitsky (1968) found leftward asymmetry (greater size on the left than the right) of the PT in two-thirds of individuals. Around the same time in the late 1960s, Juhn Wada’s test of alternately anesthetizing the cerebral hemispheres had also demonstrated the widespread left-hemisphere dominance for language processing. The implied association between leftward structural asymmetry and functional lateralization led some authors to suggest that cerebral asymmetry is a defining feature of the human brain (Corballis, 1991; Crow, 2000). In fact, there is uncertainty concerning the relationships between different measures of asymmetry and corresponding language lateralization. Individuals with situs inversus (reversal of the bodily organs) who have reversed frontal petalia (asymmetric extension of the anterior limit of the frontal lobe) still show normal asymmetry of the PT (Kennedy et al., 1999). This suggests dissociation between elements of asymmetric structure. Other researchers have found that, although PT asymmetry and language laterality are significantly left-hemisphere biased, they may not be correlated (Eckert et al., 2006).

A more complex picture has emerged from psychological and neuroimaging studies which have clarified more precise associations between structure and function. The PT may be subdivided into medial, lateral, and caudal parts, each associated with different aspects of speech processing (Tremblay et al., 2013). Anterior STG is sensitive to syntactic word category violation in a sentence (Friederici et al., 1993), while the posterior STG supports a left-hemisphere bias for phonological processing (e.g., Robson et al., 2012). Meanwhile, the right-hemisphere auditory areas are dominant for music perception in untrained listeners (Ono et al., 2011), although this functional asymmetry is modulated by degrees of expertise and ability. Therefore, the evidence for two aspects of lateralization, structural and functional, has become increasingly refined, suggesting that lateralized functions (e.g., language) often depend on multiple cognitive components (e.g., phonology, prosodic intonation etc.) that may be modular in nature and structural asymmetry (e.g., Sylvian fissure length) depends on smaller structural components (e.g., anterior, posterior STG, sub-regions of PT). The relationship between structure and function appears to depend on the lateralization of these localized components.

The search for the link between structure and function leads therefore to the small-scale modular components that constitute the functions of interest. Indeed, inconsistent matching between measures of asymmetry and lateralization may be due to attempts to match incompatible levels (e.g., attempting to match a small structural subregion asymmetry with the lateralization of a function that emerges from the interaction of multiple regions). In terms of function, two underlying processing biases are apparent at a basic level that may contribute to language laterality. First, the left hemisphere is biased toward processing short temporal transitions in the sound signal which is especially suitable for recognizing speech (Efron, 1963; Tallal et al., 1993; Shtyrov et al., 2000; Zatorre et al., 2002). Conversely, the right hemisphere is biased for spectral sound processing (Zatorre and Belin, 2001) which may form the basis of the dominance of music perception in the right hemisphere in untrained listeners. Second, evidence supports the concept that in the generation of “meaning” the left parieto-occipito-temporal junction (Wernicke’s area) is associated with the activation of more discrete, narrow, semantic associations, whereas the right hemisphere activates more distributed semantic fields appropriate to its greater sensitivity to context (Rodel et al., 1992). Event-related potentials (ERPs) in the STG are the first to diverge depending on the semantic categories of words (Dehaene, 1995) consistent with a role for this region early in category discrimination (although see Eckert et al., 2006 for consideration of an alternative – that this is a response to phonology secondary to meaning). Such ERPs are asymmetrical between the hemispheres, for example, a left temporo-parietal negativity for animal names and verbs and a left inferior temporal negativity for proper names.

What level of structural focus is appropriate to identify corresponding anatomical components underlying regional asymmetry? Not all measures of the superior temporal plane identify hemispheric asymmetries. Since the original observations by Geschwind and Levitsky (1968), Zetzsche et al. (2001) have shown that the definition of PT borders influences the detection of cerebral asymmetry. Pearlson et al. (1997) suggested that measurement of surface area is more important than volume and Barta et al. (1997) detected asymmetries by surface area measurements that were not detected by volume measures. Both are consistent with the hypothesis of Harasty et al. (2003) that asymmetry of the PT is due to lengthening of the cortex on the left side relative to the right. These measures at the surface may therefore indirectly reveal differences in the underlying neural circuitry that is the basis for differences in processing bias between the hemispheres.

The horizontal expansion of cortical surface during development (within individual brains), and across evolutionary time (between species), is largely due to the proliferation and spacing of radial minicolumns of cells that form the cortex (Rakic, 1995). These microscopic structures persist throughout the mature brain, where they span the 3–4 mm depth of the cortex with a horizontal width of approximately 50 μm. Minicolumns emerge by radial migration of cells toward the brain’s surface during embryonic formation of the cerebral cortex. Column-like radial organization is found for cell bodies and their axonal and dendritic connections. Auditory cortex in the STG develops a clear columnar cell distribution by the third trimester of fetal life, which is established in early childhood, although axonal maturation continues up to at least 12 years of age (Moore and Guan, 2001) and probably later in more associative regions. Although the human minicolumn asymmetry is not large (Buxhoeveden et al., 2001; Hutsler, 2003), it is estimated to account for a surface area asymmetry of 8–9% of the region’s size (Chance et al., 2006). Notably, this asymmetry of minicolumn spacing is absent in the equivalent areas of the brains of other apes (Buxhoeveden et al., 2001). The microscopic asymmetry in humans is also detected at the slightly larger scale of inter-connected “macrocolumn” patches (approximately 500 μm diameter) which are more widely spaced in the left than in the right auditory association cortex (Galuske et al., 2000). Recent single-unit electrophysiological recordings have demonstrated that cells within the same minicolumn share greater similarity of stimulus sensitivity than with cells in neighboring columns (Opris et al., 2012). The combination of stimulus-sensitive columns in a region presumably confers processing specialization.

Minicolumn organization in the PT has been found to correlate with cognitive scores (tests such as the Mini Mental State Exam which covers a range of tasks including object naming and simple sentence construction; Chance et al., 2011b). The relationship with cognition was specific to minicolumn measures and was not found for neuron density, as also reported in monkeys (Cruz et al., 2009). It has been suggested that greater spacing of minicolumns in human association cortex results in less-overlapping dendritic trees and allows more independent minicolumn function (Seldon, 1981a,b). This is consistent with the association between the greater surface area and the wider spacing of evoked electrophysiological activity peaks in the superior temporal plane of the left hemisphere compared with the right (Yvert et al., 2001). Harasty et al. (2003) have developed the notion that widely spaced minicolumns function as discrete units facilitating computational processing of more independent components, whereas densely spaced minicolumns permit greater overlapping co-activation and therefore confer more holistic processing. In Jung-Beeman’s (2005) model, the basal dendrites of right-hemisphere pyramidal neurons have longer initial branches and more synapses further from the soma than left-hemisphere neurons where the more widely spaced minicolumns have more dendritic branching within their territory. Wider minicolumn spacing is therefore associated with higher resolution processing across less-overlapping basal dendritic fields whereas dense minicolumn spacing is associated with lower resolution, holistic processing due to relatively greater distal sampling of more overlapping fields (Jung-Beeman, 2005).

## EVOLUTIONARY COMPARISON OF AUDITORY AND FACE-PROCESSING ASYMMETRIES

It has been suggested by some (Annett, 1985; McManus, 1985) that hemispheric asymmetries are human specific and offer a neural correlate of uniquely lateralized function, including language, in humans. A challenge to this thesis is found in comparative neuroanatomical studies that have reported the presence of asymmetries in other primate species (LeMay and Geschwind, 1975; Holloway and De La Coste-Lareymondie, 1982; Gannon et al., 1998). However, in contrast with the macroscopic picture based on surface landmarks, current evidence indicates evolutionary discontinuity for microscopic, cytoarchitectural asymmetry. Region size estimates based on cytoarchitecturally defined boundaries have found that asymmetries are weaker in chimpanzees compared to humans (Spocter et al., 2010), indicating that species differences in asymmetry are more readily identified when cytoarchitectural features are used. Hemispheric asymmetries at the neuronal level show yet more consistent differences between humans and other primates (Chance and Crow, 2007). Asymmetry in the spacing of minicolumnar units of neurons in the human PT is absent in the brains of other primates (Buxhoeveden et al., 2001), and there is a preponderance of large layer III pyramidal neurons (Hutsler, 2003) with wider dendritic arbors (Seldon, 1981a,b) filling the space in the left hemisphere compared with the right in humans. Both Broca’s area and Wernicke’s area in humans have hemispheric asymmetries of neuropil (Amunts et al., 1999; Anderson et al., 1999). Chimpanzees lack neuropil asymmetry in the equivalent areas (Sherwood et al., 2007). Neuron density in the posterior STG (area Tpt) in chimpanzees is not asymmetrical (Schenker et al., 2005). It is worth acknowledging, however, that symmetry of cytoarchitectural organization may not always be detected – Spocter et al. (2012) did not detect a significant asymmetry of neuropil fraction in the PT or Heschl’s gyrus in chimpanzees or humans.

Face processing is another highly evolved ability in primates that provides an interesting comparison in two respects – it is asymmetrically dominant in the opposite direction to language, i.e., face processing is dominant in the right hemisphere in humans (Kanwisher et al., 1997), and it is also a function successfully performed by our closest primate relative, the chimpanzee (Parr et al., 2009). Although both species perceive faces in a predominantly holistic manner (see Taubert and Parr, 2010), this process is clearly lateralized in humans in whom holistic analysis is biased to the right hemisphere (while individual facial features are detected in the left hemisphere; Rossion et al., 2000). The face processing area in the ventral temporal cortex is part of the brain network supporting social cognition in humans and other primates and is found in the mid-fusiform region (roughly equivalent to Brodmann area 37 in human brain). This area falls within a larger surrounding region that processes visual objects in general. This local specialization and the high heritability of face processing (Zhu et al., 2010) make it plausible that there is a detectable neuroanatomical correlate in this region, although the extent to which the neural structure depends on genetic contribution or early social learning is unresolved.

In humans, cells have become large and less densely packed in the evolution of mid-fusiform cortex compared to the chimpanzee and this is accentuated in the left hemisphere with the result that there is an inter-hemispheric asymmetry that is not found in chimpanzees (Chance et al., 2013). Consequently, in humans, the wider minicolumns and larger neurons are found in the hemisphere opposite to the one that is dominant for face perception. Therefore, unlike auditory language processing, it appears that the arrangement of minicolumns that confers dominance for face processing is the thinner, denser spacing that is found in the right hemisphere (Chance et al., 2013). Meanwhile, the absence of asymmetry in chimpanzees may relate to better performance than humans in tasks such as inverted face recognition that have ecological validity for chimpanzees (Matsuzawa, 2007). The human asymmetry is relatively confined to the mid-posterior fusiform region, as a previous study that included the more anterior fusiform (area 20) reported that minicolumn width in human subjects did not show a statistically significant asymmetry (Di Rosa et al., 2009). A further indication that this functional specialization is associated with minicolumn structure – face discrimination ability is reduced in old age (an effect described as “dedifferentiation”; Goh et al., 2010) and marked minicolumn alteration is also found in fusiform cortex in old age (Di Rosa et al., 2009). As with auditory language processing, there is also left-hemisphere dominance for written language and disordered reading is associated with damage to the left temporo-parietal area (the angular gyrus), first noted by the 19^th^-century neurologist Dejerine. However, inability to read (“pure alexia”) is associated with damage to the left mid-fusiform gyrus (Leff et al., 2006). It has been suggested that the wider minicolumn spacing in this region of the left hemisphere may relate to its role in visual word recognition in humans in addition to its role in face processing (Chance et al., 2013).

## MECHANISTIC MODELS

If the point of convergence between functional and anatomical lines of evidence implicates these small, modular units, a mechanistic model is desirable to explain this across different domains of processing. In the visual domain, it is possible that wider minicolumn spacing may be associated with detailed feature processing, whereas thin minicolumns may facilitate holistic, configural processing of the type usually associated with face processing. In such a scheme, face processing is similar to music processing. Holistic, configural processing for face recognition (or music) benefits from the computational overlap generated by densely spaced minicolumns in the fusiform gyrus. This mechanistic interpretation is consistent with a correspondence between the rightward lateralization of holistic face processing and the thin minicolumns found in the right hemisphere in humans and replicates the structure–function correspondence found in the auditory domain although the processing demands of the function lead to different hemispheric dominance. This suggests that minicolumn width is dissociated from “dominance,” *per se*, and instead relates to the type of processing: featural or holistic. The wider minicolumn spacing in the left STG facilitates fine temporal discrimination because minicolumns function as more discrete computational elements, whereas dense minicolumn spacing in the right STG supports broad spectral processing, due to the minicolumns’ greater computational overlap. The hemispheric processing bias for a given task is likely to depend on the degree to which task success emphasizes local or global processing and the hemispheric asymmetry of minicolumnar units in the brain region associated with that functional domain. This concept refines the simple notion that a larger brain area is associated with dominance for a function and offers an alternative, mechanistic explanation associated with “processing type” (Van Veluw et al., 2012).

The processing-type hypothesis has the advantage of acknowledging the active role of the “non-dominant” hemisphere. It is recognized increasingly that many tasks combine elements of both holistic and featural processing (Rossion et al., 2000). Thus, two streams of processing occur in parallel – global processing in broad-activation fields of the right hemisphere and local processing in focused fields of the left hemisphere. In isolation, these streams simply encode two separate levels of detail, but by cross-referencing the differences between the active fields of the two hemispheres via the corpus callosum the relationship of local features to global features may be encoded. The emergent hierarchy of features within features is a recursive structure that may functionally contribute to generativity – the ability to perceive and express layers of structure and their relations to each other. It has been argued that recursive generativity is an essential, or even, the key component of human language behavior (Crow, 2005). The description here is consistent with such a scenario although it cannot be concluded that the presence of recursion necessarily entails this form of structural asymmetry. Cytoarchitectural asymmetries have been found in normal auditory cortex that correlate with the number of axons passing through the connecting regions of the corpus callosum (Chance et al., 2006). A greater number of minicolumnar units in the hemispheric region that is typically functionally dominant was associated with more inter-hemispheric connections through the area of the corpus callosum connected to that region.

This mechanistic, processing-type hypothesis potentially contributes to a coherent, descriptive account of cerebral asymmetries of structure and function. However, it is also necessary to identify an evolutionary advantage conferred by this organization, particularly if it is different in humans from other apes. Although not originally associated with asymmetry, Gabora (2002) has proposed a model of the evolutionary enhancement of cognitive processing capacity in humans through the cross-referencing of different levels of conceptual organization. Similar to the recursive process described above, Gabora (2002) describes the interpolation between concepts at “varying levels of abstraction (i.e., cup, container, thing)” as providing stepping stones in a recursive process of “variable focus,” She speculates that a pre-palaeolithic mind “activated regions of conceptual space of fixed size with limited ability to focus,” but the capacity for variable focus evolved enabling alternately widening and narrowing the “activation function.” Although Gabora (2002) describes this as a process of focus fluctuating over time, at least part of this requirement may be met concurrently by the asymmetry between hemispheres as they process different levels of abstraction. Furthermore, although Gabora’s (2002) “activation function” was not clearly defined, it seems reasonable to interpret it not just in the abstract but as a field of activated units such as the overlapping minicolumns described above.

## PSYCHOLOGICAL SPACE AND LATERALIZED PROCESSING

The Gabora’s (2002) model suggests an evolutionary benefit that may be provided by different levels of processing, compatible with existing lateralized processing biases. The proposed advantage of variable focus is to expand the capacity of conceptual space by interpolation between concepts. In statistical terms, it is equivalent to the generation of continuous data rather than categorical data. In psychological terms, it may be described as the contrast between dimensional and categorical processing. Therefore, if the mechanistic interpretation of microstructural asymmetries is related to this interpolation between concepts and therefore to the generation of continuous dimensions that define a continuous conceptual space, one would expect some association with the organization of the dimensions of conceptual space in the two cerebral hemispheres.

It is often challenging to obtain data for the separate hemispheres, however, in the language domain some investigations have provided data on the organization of semantic space in each hemisphere. Taylor et al. (1999) found that the right hemisphere uses more dimensions than the left hemisphere to represent the semantic map in typical subjects. It is unclear if more dimensions constitutes less efficient coding (i.e., in the right hemisphere each dimension may contribute less to the representation of different concepts, whereas in the left hemisphere the dimensions are more discriminatory and so fewer are needed) or more complex representation (i.e., the right hemisphere may take account of more aspects of a given concept). However, more diffuse activation of the network in response to a linguistic stimulus, consistent with the model of holistic, overlapping activation described above, has been proposed to explain the lesser discrimination between primary and secondary word meanings that is also typically found in the right hemisphere (Weisbrod et al., 1998). This lower resolution discriminative capacity in the right hemisphere is found for face processing even as the right hemisphere is also dominant for making categorical (face vs non-face) distinctions (Meng et al., 2012). This is consistent with the notion that the holistic processing of the densely spaced minicolumns in the right hemisphere facilitates broad categorical processing, whereas the left hemisphere differentiates components within dimensional psychological space. The combination of an increased number of dimensions and more diffuse activation in the right-hemisphere network suggests that the dimensions are partly correlated and less separable than truly orthogonal dimensions.

The phenomenon of key dimensions along which concepts can be organized provides a structure for mentally sorting concepts. This is desirable so that semantic information may be efficiently processed at different levels of elaboration (Craik and Lockhart, 1972). Similar to Gabora’s (2002) variable focus, a benefit may be conferred by complementary forms of elaboration with one hemisphere emphasizing the clear separation of concepts and the other allowing more overlap. Different metrics underlying the conceptual space are possible (Gardenfors, 2000), which suggest differences in conceptual organization corresponding to hemisphere differences. Just as with the revolution in understanding of the physical universe in the early 20th century, which indicated that physical space is curved, there have been suggestions that the underlying structure of conceptual space is also not what we may first assume. For example, various psychological spaces are better represented by the “city-block” metric (Arabie, 1991) rather than the familiar Euclidean metric that has been typically assumed (e.g., in multi-dimensional scaling analysis such as Paulsen et al., 1996). The metric is so-called because the distance between concepts is measured as if restricted to a grid-like system of roads (hence “city-block” or “Manhattan” metric) rather than “as the crow flies” in Euclidean space. In the city-block metric, points equidistant from a central point lie on a square around it rather than a Euclidean circle. It has been argued that the sharp-cornered form of the non-Euclidean city-block metric better models the natural tendency to perceive discontinuities between concepts with the corners of a square creating a discontinuity between the concepts on either side of them (Arabie, 1991; Gardenfors, 2000). The orthogonal edges of the square mimic the way conceptual dimensions (such as “size” and “domesticity”) are not arbitrary and interchangeable. The difference between hemispheres in the separation and correlation between dimensions suggests a hemispheric difference in the metric of the conceptual space.

The separation of conceptual dimensions also changes during development. Normally, a developmental shift occurs: whereas older children and adults perceive dimensions such as high and tall, or big and bright, to be separable, young children tend to confuse these concepts (Carey, 1978). Goldstone and Barsalou (1998) have described the development of reasoning about dimensions: “dimensions that are easily separated by adults, such as the brightness and size of a square, are treated as fused together for children… [they] have difficulty identifying whether two objects differ on their brightness or size even though they can easily see that they differ in some way. Both differentiation and dimensionalization occur throughout one’s lifetime.” This has been described as a developmental shift from a more Euclidean cognitive metric to the more separable dimensions of the city-block metric (Gardenfors, 2000). The development of more orthogonal dimensions therefore is associated with more sophisticated cognitive discriminative ability. Aspects of brain structural maturation and plasticity presumably relate to this process of cognitive maturation. The increase in discrimination associated with orthogonal dimensions is similar to the acquisition of expertise, which is often associated with left-hemisphere specialization for fine-grained difference judgements, e.g., for faces, word meaning and music. The process, extended over childhood, is also likely to be influenced by the social and cultural environment, including the requirements of social integration and communicative pressure for shared conceptual frameworks. Appropriately, it is the same hemisphere (the left) that is associated with the acquisition of expert discrimination and dominance for the communicative faculty of language that reinforces it.

## SCHIZOPHRENIA AND AUTISM

Testing the mechanistic role of cytoarchitectural asymmetry on these aspects of cognitive function is challenging as the lateralized functions of interest appear to be confined to humans and, debatably, few other animals. However, disruptions of both minicolumnar structural organization and lateralized function are found in human neuropsychiatric disorders which provide further insight.

Altered cerebral asymmetry has been found in schizophrenia (Bilder et al., 1994; DeLisi et al., 1997; Chance et al., 2005) and the prominent role of language anomalies in schizophrenia also implicates lateralization (Crow, 1990). The auditory region offers one of the clearest associations between psychotic symptoms and brain structure, as it is activated during auditory hallucinations (Shergill et al., 2000; Ropohl et al., 2004). The loss of left-hemisphere ERP mismatch responses to anomalous words at the end of a sentence, based on incongruous word meaning (Spironelli et al., 2008), provides a link between the sensory, phonological abnormalities and linguistic meaning. Reduced gray matter in this area, including the PT, is one of the most replicated structural changes in the disorder. Minicolumn asymmetry of this region is also altered in male patients (in whom illness is usually more severe) in such a way that both hemispheres are configured more like the typical right hemisphere (Chance et al., 2008).

Word generation (semantic fluency) tests the integrity of the semantic network that encodes basic knowledge about the meanings of words. Patients with schizophrenia have been shown to have networks that are less organized than those of control subjects (Paulsen et al., 1996; Rossell et al., 1999). If increased number of dimensions is taken to be indicative of more diffuse activation in the right-hemisphere network in normal subjects (as described above; Taylor et al., 1999), then the hypothesis that patients have unusually diffuse semantic associations in the left hemisphere as well as the right hemisphere (Weisbrod et al., 1998) predicts that patients use more, poorly discriminative dimensions overall. This is supported by several studies which reported less effective mapping of semantic space in low dimensions for schizophrenia, indicating the requirement for more dimensions (Paulsen et al., 1996; Rossell et al., 1999). The evidence that semantic category boundaries are less clear in schizophrenia (Paulsen et al., 1996) raises the prospect that the city-block metric may not provide a better fit for patients. In adolescent onset schizophrenia it has been found that the city-block metric provided a less beneficial data fit than in controls (Chance et al., 2011a). Therefore, alterations in the dimensions of conceptual space, consistent with disruption of lateralized cognitive processing biases, accompany abnormal anatomical structure of the cortex, including altered asymmetrical cytoarchitecture in schizophrenia.

The developmental shift from the Euclidean cognitive metric to the more separable dimensions of the city-block metric proposed by Gardenfors (2000) may be relevant in the neurodevelopmental context of schizophrenia. Although there is a clear genetic component in the etiology of schizophrenia, onset of illness is not identified until adolescence or early adulthood. It has been proposed that, structurally, this may be linked to the time course of myelination (Crow et al., 2007; Chance et al., 2008). Functionally, it may be linked to the shift in cognitive metric and as dimensionalization matures the anomalies associated with psychosis are exposed, leading to the recognition of “onset” and diagnosis.

Schizophrenia patients sometimes have difficulty in recognizing their own face (Kircher et al., 2003) and minicolumns have also been shown to be altered in the fusiform gyrus in patients (Di Rosa et al., 2009). In another neuropsychiatric condition, people with autism have a selective deficit in perceiving facial expressions categorically (Teunisse and de Gelder, 2001) which affects activation of the fusiform gyrus (Pierce et al., 2004). One of the few neuropathological features of the disorder is altered minicolumn organization (Casanova et al., 2006) accompanied by altered neuron density in layer III of the fusiform gyrus (Van Kooten et al., 2008). Although it is not, so far, apparent that the effect in autism is asymmetrical between the hemispheres, it is clear that these alterations present a risk of disruption to the very structures that support lateralized face processing and are consistent with atypical processing in that functional domain. Indeed, attempts to characterize the deficits in ASD at a broader level led to the “weak central coherence” hypothesis (Frith, 1989) which proposes that the core difference in ASD involves poor integration of “featural” information into a coherent whole.

In terms of language and theory of mind, autism is associated with excessively literal interpretation of word meaning and under-interpretation of social relevance at the pragmatic level. Both appear to emerge from a disruption of the ability to interpret layers of meaning and their relations to each other. Altered processing of semantic categories has been implicated in autism (Gastgeb et al., 2006; although further studies have suggested that the effects are often subtle). More broadly, in visual categorization tasks, deficits in prototype formation have been indicated (Gastgeb et al., 2012) and altered influence of categorical knowledge in autism has been interpreted as a reduction of top-down influence on perceptual discrimination (Soulières et al., 2007). In the context of altered minicolumn structure, these effects are consistent with the mechanistic model of minicolumn asymmetry influencing different levels of processing that are lateralized for some functions.

In contrast to autism, schizophrenia is associated with over-interpretation of word meaning at the semantic level and over-interpretation of relevance at the level of pragmatic competence, Altered interhemispheric connections have been found to be correlated with minicolumn asymmetry in auditory language cortex in schizophrenia suggesting a link to language-processing anomalies that occur in the disorder (Chance et al., 2008; Simper et al., 2011). Therefore, both disorders may involve a contribution from disequilibrium in the processing of local and global features related to the disorganization of minicolumnar units of processing.

## Conflict of Interest Statement

The author declares that the research was conducted in the absence of any commercial or financial relationships that could be construed as a potential conflict of interest.
